# Relationship between stress levels and coping techniques in caregivers of children with autism spectrum disorder in Chile, 2021

**DOI:** 10.1590/1980-5764-DN-2022-0112

**Published:** 2024-08-26

**Authors:** Bárbara Cerda-Aedo

**Affiliations:** 1 Universidad Adventista de Chile, Dirección de Posgrado e Investigación, Chillán, Chile.; 2 Universidad Internacional de la Rioja, Logroño, España.

**Keywords:** Autism Spectrum Disorder, Caregivers, Adaptation Psychological, Estrés Psicológico, Neuropsychology, Transtorno do Espectro Autista, Cuidadores, Adaptação Psicológica, Estresse Psicológico, Neuropsicologia

## Abstract

Today, talking about autism spectrum disorder (ASD) is the same as talking about cases that occur in one in 160 births worldwide. Some of them will be able to live independently when they grow up while others will have less autonomy and will be more dependent, requiring the support of caregivers throughout their lives.

**Objective:**

Understanding the emotional burden that this could generate on parents, we sought to analyze the level of stress and coping techniques in caregivers of children with ASD in Chile, 2021.

**Methods:**

Interview with a sample composed of 61 parents or guardians of people with ASD.

**Results:**

After data analysis, it was possible to perform a statistically significant correlation (p=0.002) between the level of stress and the coping strategies (problem-solving, self-criticism, emotional expression, wishful thinking, social support, cognitive restructuring, problem avoidance, and social withdrawal). In addition, positive strategies that reduce stress levels in parents or caregivers of children with ASD were identified (problem resolution, cognitive restructuring, social support, and emotional expression).

**Conclusion:**

Through this research, it was possible to respond to each of the stated objectives, managing to determine what were the characteristics of caregivers and their main difficulties. It was also observed that the majority lost the possibility of working to dedicate themselves to the care of the diagnosed person.

## RESUMO

Hoje, falar sobre transtorno do espectro autista (TEA) é falar sobre casos que ocorrem em um a cada 160 nascimentos em todo o mundo. Alguns deles conseguirão viver de forma independente quando crescerem, e outros terão menos autonomia e serão mais dependentes, necessitando do apoio de cuidadores ao longo da vida.

**Objetivo:**

Entendendo o gasto emocional que isso pode gerar nos pais, buscamos analisar o nível de estresse e as técnicas de enfrentamento em cuidadores de crianças com TEA no Chile, 2021.

**Métodos:**

Entrevistar uma amostra composta de 61 pais ou responsáveis de pessoas com TEA.

**Resultados:**

Isto nos permitiu obter, após a análise dos dados, uma correlação estatisticamente significativa (p=0,002) entre o nível de estresse e as estratégias de *coping* (resolução de problemas, autocrítica, expressão emocional, *wishful thinking*, apoio social, reestruturação cognitiva, evitação de problemas e retraimento social). Além disso, foram identificadas estratégias positivas que reduzem os níveis de estresse em pais ou cuidadores de crianças com TEA (resolução de problemas, reestruturação cognitiva, apoio social e expressão emocional).

**Conclusão:**

Além disso, esta investigação conseguiu responder a cada um dos objetivos enunciados, conseguindo determinar quais eram as características dos cuidadores e quais as suas principais dificuldades. Observar-se-á também que a maioria perdeu a possibilidade de trabalhar para se dedicar ao cuidado da pessoa diagnosticada.

## INTRODUCTION

To better comprehend the term of *autism spectrum disorders* (ASD), it is important to understand that it refers to a group of conditions "characterized by some degree of difficulty with social interaction and communication. Other characteristics are atypical patterns of activities and behaviours, such as difficulty with transition from one activity to another, a focus on details, and unusual reactions to sensations"^1^. ASD tends to emerge during infancy and persists into adulthood^2^. Nowadays, it occurs in one out of 160 births globally. Some of them, when reaching adulthood, manage to live independently; others, however, remain under life-long care administered by their parents^3^. Regarding global numbers of ASD, it must be kept in mind that cases can present variations between investigations^1^.

It is difficult to specify the impact of ASD on a local level because in Chile there are no studies that account for the prevalence of this illness. However, when taking into consideration the international prevalence of the disorder, which is nine for every 1,000 live births, and cross-referencing it with national data on birth rates in the country (240,569 live births in 2007), it can be inferred that there are approximately 2,156 children that could be diagnosed with ASD in Chile^4^. It is an alarming number, especially if factors that go further beyond the disorder itself are considered; for example, parental stress associated with the rearing of children with ASD.

According to Abidin^5^, parental stress is understood as the total stress that parents experience as a result of the innate characteristics of their children, considering the innate characteristics of the parents. In the case of parents or caregivers of children with ASD, they have to deal with obligations related to the conditions of their children's disorder in addition to those common ones required for parenting, which generates high levels of stress^6^. This stress, on top of generating changes at the psychological level, negatively affects the immune system and leads to difficulties in socialization and relationships, and also in childrearing. All of this could have a significantly negative impact on the child, such as an increased risk of suffering abuse or negligence^7^.

Moreover, the literature on the subject is clear in showing the negative impacts of high levels of parental stress on the mental health of their children, as this stress has been associated with an increased and varied clinical symptomatology of children^8^. Thus, from a professional point of view, it is understood that reducing stress levels in parents is necessary not only for the well-being of the parents/caretakers themselves, but also for the children. Consequently, this also results in a necessity to highlight coping mechanisms or strategies as a mediating and supportive element for those in charge of children with ASD.

Lazarus & Folkman in 1986^9^ proposed the concept of coping strategies, defining it as efforts and resources at a cognitive and behavioral level which a person applies in order to cope with stress factors and, in some manner, subdue the emotional state produced by high levels of stress. These strategies are understood as the dynamic process in which, through the evaluation of the situation in which a person lays out their own resources, they are able to cope with the issue at hand^10^. According to Lazarus & Folkman^9^, they could be classified in two big groups:

- Strategies centered on the problem; and
- Strategies centered on emotions.

With the former, the person gathers information regarding what they are able to do, and then proceeds with an intent to modify the situation causing stress. In the latter group, the person confronts the situation causing stress by regulating the emotions produced by it^10^.

The person then uses these strategies to cope with high levels of stress, which may or may not be adaptive^11^. This is the motive for which it is of great importance to know, from the professional point of view of a neuropsychologist, which strategies should be better favored when dealing with parenting-related stress. Taking all of this into consideration, this study sought to analyze whether there is a statistically significant relationship between the types of coping strategies and the stress level of parents or caregivers of children with ASD in Chile. This could help to orient and support parents and aid them in choosing adaptive strategies, lowering their emotional burdens, so they may better care for their children diagnosed with ASD.

## METHODS

### Quantitative and cross-sectional analysis design

The sample was composed of 61 direct caregivers (parents, grandparents, siblings, and others) of at least one person with ASD. These people lived or have lived for over a year in Chile. For the purpose of this study, we worked with caregivers that participated with the organization MiTea during the process of research, in Chillán, Chile, and with caregivers found in Facebook groups dedicated to ASD (Table 1) at the same time. The exact number of study people in each group is unknown, as the process involved total anonymity for both the researcher and the participants.

**Table 1:** Facebook groups from which the samples were collected.

Group name	Number of members	Type of group
Espectro Autista (TEA) Chile Unido	207	Public
Espectro Autismo Chile	10 thousand	Private
Autismo Tea Grupo	8,1 thousand	Private
Junta TEA Chile en General	2,7 thousand	Public

Abbreviation: TEA (in Spanish): autism spectrum disorder.

The sample was obtained through random cluster sampling. The criteria for inclusion were the following: the caretaker must have lived in Chile for over a year, or be Chilean and be the direct caretaker of the person with ASD; and the person with ASD must be 3–22 years of age and decide to participate in the study voluntarily. On the other hand, the criteria for exclusion were that the participants wanted to retire from the project at any given time, that they did not complete the survey or decided not to respond, or that they did not send the informed consent forms in the time given.

For the process of data collection, three automated surveys were utilized, two of which were oriented toward the variables of stress and coping, while the other was prepared to measure the sociodemographic origin of the participants.

### Sociodemographic survey

This survey was composed of 16 questions, some oriented to personal and work-related aspects of the caregivers, and others to the neuropsychological aspects of the person with ASD. The survey was created by researchers and validated by professors specialized in the area. It was based on Autism Speaks’ Caregiver Needs Survey, developed by Amy Daniels and the National Coordinators of the Southeast European Autism Network (SEAN)^12^. Moreover, questions which were considered necessary for the population sample was also added. The survey did not include appropriate scales as it only sought to provide a description at a sociodemographic level for the sample and not a statistical analysis of it. The survey had qualitative and quantitative questions.

### Coping strategies inventory, adapted

An analytical survey was applied, made up of 40 questions, both positive and negative, to evaluate the dimensions of coping. As being a Likert-type inventory, it has scoring ranges from 0 to 4, where 0=Absolutely and 4=Entirely.

Regarding the dimensions measured by the survey, these were divided into eight issues: problem-solving (PS), self-criticism (SC), emotional expression (EE), wishful thinking (WT), social support (SS), cognitive restructuring (CR), problem avoidance (PA), and social withdrawal (SW). In order to calculate each dimension, the scores of all respective questions had to be added so as to obtain the natural scores as a result. In addition, this survey had the keys for converting natural scores to the corresponding scales.

### Stress symptomatology inventory

The Stress Symptomatology Inventory (ISE in Spanish) is an analytical survey containing 30 items, all of which are negative. Each question is scored from 0 to 4, with 0=Never and 4=Assiduously.

The choice of the ISE as an instrument for assessing stress in adults was based on its advantages over other tools and its validation in the target population. Among the advantages of this inventory is the specificity to evaluate the level of stress, addressing physical and emotional aspects, as well as its convertibility to be applicable to the Chilean population, which due to its extensive territory, can vary in important aspects between sectors. These aspects are not a problem when an instrument such as the ISE is used.

Furthermore, the evidence of its reliability and validity supports the decision for this tool over others that evaluate the same variable. These attributes justify the choice of the ISE as an applicable instrument for the evaluation of stress in the specific framework of the research.

In order to initiate the research work, it was sent to the ethics committee of the International University of La Rioja, Spain, which evaluated and approved the project. In addition, a template was produced for Informed Consent to be signed by participants before commencing data collection. This consent assured the protection of the information obtained from the sample and allowed them to participate voluntarily.

The results were primarily described using descriptive statistic. For the quantitative variables, averages and standard deviations were used, and for the qualitative variables, frequency and percentage tables.

Regarding the statistical analysis, Spearman's test was implemented to determine the correlation between the variables. In this case, the scores from the Stress Symptomatology Inventory and Coping Strategies Inventory, and the eight dimensions of the Coping Strategies Inventory were applied. In order to establish a relationship between the variables, a value of p>0.05 was considered.

The analysis of the data (with scales) related to the Coping Strategies Inventory was performed and the scores from those dimensions that were negative were inverted to transform them into positives. In this way the final analysis between the variables of stress and coping strategies was not influenced by negative scores which would have given high scores, making a correct analysis to correlate or discard the established hypothesis impossible.

## RESULTS

In the following text, the results obtained from a group of caregivers for people with ASD surveyed in Chile during the year 2021 will be presented.

With regards to the place of residence of the study participants, it was observed that 100% of the population was Chilean. This is emphasized due to the fact that this aspect was an important criterion for inclusion in participating in the research (Table 2).

**Table 2:** Sociodemographic aspects of the sample.

Sociodemographic aspects		Frequency	Percentage
Chilean residents		61	100.0
Relationship	Mother	60	98.4
	Stepfather	1	1.6
Remunerated job	Yes	31	50.8
	No	30	49.2
Someone else contributes financially to the family	Yes	54	88.5
	No	7	11.5
There is more than one person in the family with ASD diagnosis	Yes	12	20.0
	No	48	80.0
Current diagnosis of the person with ASD	ASD	42	68.9
	Autism or Autistic Disorder	10	16.4
	PDND	2	3.3
	Asperger syndrome	7	11.5
Sex of the person with ASD who is in your care	Female	19	31.1
	Male	42	68.9

Abbreviations: ASD, autism spectrum disorder; PDND, pervasive developmental disorder not otherwise specified.

Regarding the relationship of the caregiver with the ASD child, it was noted that 98.4% of the caregivers were mothers, followed by stepfathers (1.6%). It is important to realize that fathers were not present in the study (Table 2).

Of the sample's participants, 50.8% reported having a paid job, while 49.2% related not having. It was identified, however, that 88.5% of the families received financial support from another family member.

Another important sociodemographic aspect was the diagnosis of more than one relative (20%). Most of the families screened (80%) showed that, besides the one person with ASD, no one else in the family had such disorder (Table 2).

The sex of the majority of people diagnosed with ASD was male (68.9%). As to the level of language development, it was observed that 39.3% of children were able to use complete, complex sentences, 24.6% could not speak at all, and 16.4% could use sentences of four or more words (Table 2). Regarding the language level of children with ASD as referenced by their parents or caregivers, the majority (24.6 %) did not speak (Table 3)

**Table 3:** Level of language development of the person with autism spectrum disorder under care, according to caregivers in Chile, 2021.

Level of language development of the person with ASD under care	Frequency	Percentage
Does not speak	15	24.6
Makes use of single words (e.g., "bubbles")	5	8.2
Uses sentences made up of two or three words (e.g., "Shoes, daddy")	7	11.5
Uses sentences made up of four or more words (e.g., "I want more juice.")	10	16.4
Uses complex sentences (e.g., "I'm tired and want to sleep.")	24	39.3
Total	61	100.0

Abbreviation: ASD, autism spectrum disorder.

Among those who initially diagnosed the illness, 62.3% were neurologists, and 23.0% were from an interdisciplinary team of professionals, both counting with the majority of the scores. Psychiatrists, psychologists, and pediatricians scored 4.9, 8.2, and 1.6%, respectively.

Regarding visual contact and stereotypes, 59% of caregivers stated that sometimes the person with ASD made visual contact, 34.4% said that they did made eye contact, and 6.6% said they did not make eye contact at all. ASD people who presented stereotypes constituted 52.5%, while those who sometimes presented, made up 34.4%, and those who did not present at all, made up 13.1%.

It was reported that 54.1% of those diagnosed with ASD had never self-harmed, however, 45.9% stated otherwise, indicating that they had self-harmed in some manner.

According to the ISE, the majority of parents or caregivers of children with ASD presented high levels of stress (91.8%) (Table 4).

**Table 4:** Stress level in parents or caregivers of people with autism spectrum disorder.

Stress level	Frequency	Percentage
Low	1	1.6
Half	4	6.6
High	56	91.8

In Table 5, the analysis of the relationship between the results of the Stress Symptomatology Inventory and the Coping Strategies Inventory was demonstrated, where statistically significant correlations can be found between them (p=0.002; r=-0.392).

**Table 5:** Spearman's Rho for scores of the Stress Symptomatology Inventory and the Coping Strategies Inventory.

Spearman's Rho		Coping strategies inventory (BAREMO)
Stress symptomatology inventory	Significance (bilateral)	0.050
	Number	61
	Correlation coefficient	-0.392
	Significance (bilateral)	0.002
	Number	61

**Figure 1:**
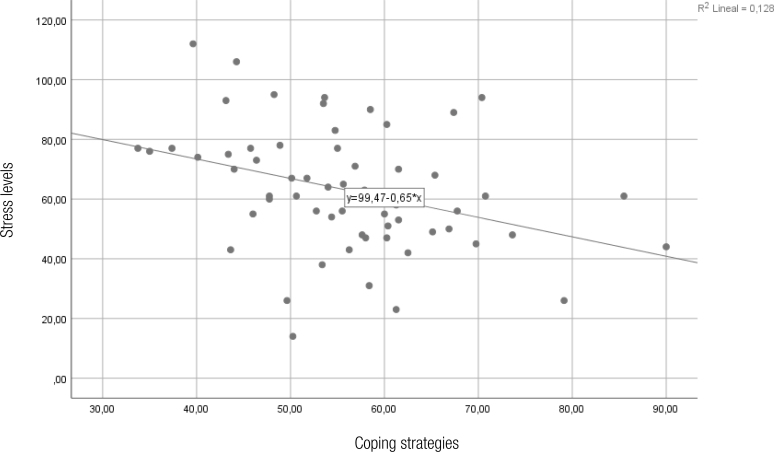
Variable dispersion graph of the stress levels and the coping strategies of people with autism spectrum disorder in Chile, 2021.

Moreover, a dispersion graph (Figure 1) is displayed in which the relationship between the numerical variables corresponding to the Stress Symptomatology Inventory and the Coping Strategies Inventory can be seen. An inversely proportioned correlation can be noted, indicating that with the increase of positive coping strategies, a decrease in stress levels in caregivers is observed. This can be seen in the lines of tendencies that indicate a relationship between the points on the graph.

Lastly, regarding the correlation between the dimensions of the Coping Strategies Inventory and the Stress Symptomatology Inventory in caregivers, it was evidenced that dimensions belonging to negative coping strategies such as SC (p=0.009), WT (p=0.006), and SW (p=0.003) had a higher correlation with higher levels of stress; whereas, of those positive dimensions, only two kept correlations with stress levels, which were EE with a negative correlation (p=-0.018) and CR with a positive correlation with stress levels (p=0.033, see Table 6).

**Table 6:** Correlation between the dimensions of the Coping Strategies Inventory and the score of the Stress Symptomatology Inventory. Sample composed of 61 parents/caregivers.

		Stress symptomatology inventory
Problem solving	Correlation Coefficient	-0.031
	Significance (bilateral)	0.812
Self-criticism	Correlation Coefficient	0.332
	Significance (bilateral)	0.009
Emotional expression	Correlation Coefficient	-0.018
	Significance (bilateral)	0.891
Wishful thinking	Correlation Coefficient	0.349
	Significance (bilateral)	0.006
Social support	Correlation Coefficient	-0.089
	Significance (bilateral)	0.496
Cognitive restructuring	Correlation Coefficient	-0.273
	Significance (bilateral)	0.033
Problem avoidance	Correlation Coefficient	0.041
	Significance (bilateral)	0.753
Social withdrawal	Correlation Coefficient	0.373
	Significance (bilateral)	0.003
	N	61

## DISCUSSION

Regarding the characteristics revealed in the studied sample about the relationship between the caregiver and the person with autism, it was found in an Ecuadorian research paper from 2020^13^, that among almost all surveyed population, the mother had to leave her job in order to take care of her child. This indicates that in the majority of the sample, the maternal figure is the one who takes care of the ASD person. This fact is coherent and in line with the result of the present investigation, where the sample shows that it is the mother who often has a direct relationship with the person with ASD.

In addition, an inversely proportional relationship was noted between the scores of the Stress Symptomatology Inventory and the Coping Strategies Inventory, which confirms the notion that coping strategies permit lowering stress levels in caregivers, lessening symptoms and potential illnesses that can develop during the process of taking care of ASD children. In this manner, a bibliographical review is coherent with this correlation, indicating that in light of the relation between these variables, the labor of health professionals is essential to detect stressors and provide positive strategies, preventing problems associated with inadequate family coping techniques^14–17^.

Another relevant issue related to what has been shown is the lack of paid work for parents/caregivers. An investigation conducted in Madrid^18,19^ showed that one of the strategies the family uses to take care of a person diagnosed with ASD includes quitting their jobs, which allows them to have more time to dedicate to the child. In the case of the current study, it is important to highlight the relationship with this task, once close to half of the population is not working; which may be due to the need to attend the person with ASD full-time. Still in the studied sample, most families have at least one person in charge of working, which brings income and resources to the family.

It is worth noting that, for genetic reasons, it is highly probable that in a family, more than one person exhibits the ASD diagnosis. According to a longitudinal study conducted by Lee et al.^20^, the probabilities of having another child with autism increase up to 18.7%, the highest risk percentage being in males. Therefore, the evidence is clear when showing that genetic factors are the main cause of children born with ASD. Besides, more than just the possibility of having more than one child diagnosed with ASD in the same family, there also exists the possibility that one of the parents or relatives has this disorder^21^.

When comparing this data with the present study, it can be observed that a high percentage of the existence of other relatives with ASD is not shown, which can be due to the lack of information or diagnoses.

Regarding the sex of the person who presents the diagnosis, the study suggests that the majority is male; corroborating the investigations in the area which indicate that the probability of ASD is higher in men than in women^22–24^.

Another important topic is the language development of people suffering from ASD; they usually experience difficulties communicating or interacting socially, as for many of them, language is a difficult form of communication^25^. This information does not correlate entirely to what was obtained in this study, in which, according to caregivers, the majority of people under their care use complex sentences to communicate.

People presenting this disorder tend to avoid visual contact and also avert their gaze, which according to new studies, may be influenced by genes^26^. Regarding the information obtained from the tutors in this investigation, most of those who are under care only directly look at people's eyes in some cases, while a very low percentage of them do not make any visual contact at all either with their family or professionals. Among the symptoms shown by the surveyed population, stereotypes stand out, identified in great part by their caregivers; in this sense the literature points toward rhythmic and repetitive manifestations such as walking on tiptoes, turning about, balancing their bodies, hitting themselves or other people^27^. However, these can be effectively treated pharmacologically, especially when their behavior becomes difficult and disruptive in intrafamilial discourse, in schools, or during medical checkups^27^. Another problem is self-harm; at least half of the caregivers have observed in their charges. Though there is currently no explanation for why people with autism cause self-harm, it is known that this could be linked to stress levels, biological predispositions, atypical sensorial processing, and even be associated with alternate methods of communication, medical problems, as well as limitations in regulating emotions, among other things. These are cases and situations that caregivers of people with ASD must face and resolve, for which education and adequate professional guidance are vital to avoid complications regarding care management^28^.

Therefore, a literature review showed that the main positive family coping strategies in ASD should include: searching for information, family integration, positivity, creating forms of communication, interaction, and cooperation^14^. Strategies that are related to each other and explain the positive correlations found in the population among the dimensions of the Coping Strategies Inventory are: problem-solving, social support, cognitive restructuring, emotional expression, and cognitive restructuring.

Based on the items of the negative dimensions of the Coping Strategies Inventory, manifestations such as the following are highlighted: feeling of guilt, willing that the situation was not happening or somehow ended, self-criticizing for what has happened, hoping that they never again find themselves in that kind of situation, hoping to have been able to change what has been happening, wishing things could have been different. Negative strategies, similar to the ones described in a literature review that has been recently mentioned, include: suppression of activities, increasing protection, negation, and social isolation^14^. The symptomatic manifestations that the reviewed literature takes into account are often associated together and explain positive correlations, which are found in the dimensions of the coping strategies instrument: self-criticism, wishful thinking, social withdrawal, and problem avoidance.

Thus, the literature indicates that stress levels in these families are superior to those families without ASD members^29^ which is coherent with what was observed in this study, since the stress levels of the caregivers, according to the inventory, had a prevalence of over 90%. At the same time, other studies detail the appearance of depressive traits with growing severity in their manifestations according to the severity of the disorder^30^.

In conclusion, regarding the hypothesis of this work and the results obtained, it was possible to determine what were the stress levels of the caregivers and their coping levels as well. That allowed the researches to create a profile of the positive and negative coping strategies.

The present study has some limitations that must be considered when interpreting its results. Adopting a cross-sectional design hinders the establishment of causal relationships between the strategies and the level of stress. Furthermore, by using convenience sampling, the generalizability of the results could be affected.

It is important to highlight that due to COVID-19 pandemic issues, the surveys had to be done virtually, which could indicate a significant bias, resulting from responses that can often be social desirability. These limitations point out the importance of expanding the field of study on parents or caregivers, in order to cover a larger population and explore those variables that were not present in this study.

The relationship between coping strategies and level of stress in parents or caregivers of people with ASD contributes significantly to knowledge. First, it allows the identification of behavioral patterns of parents or caregivers and how these are associated with their level of stress in parenting. This gives access to valuable information for the creation of development proposals, and public policies, among others, aimed at caregiver mental health.

Relating coping strategies with level of stress also allows progress to generate specific support for parents, who usually neglect their health to prioritize that of their children. Family interventions or treatments could also be carried out to help them understand how the coping strategy used can increase or decrease stress levels.

Adding these results to the existing literature can enrich the global understanding of the factors that contribute to the well-being of these families, opening lines of research-oriented to this field.

For the future, it is hoped that this study could open additional venues of research oriented toward the relief of caregivers of ASD people. As has been observed throughout this work, they present high levels of stress and coping strategies that they have been adjusting based on the few tools and knowledge they possess. It would be important to use this research to generate spaces in health centers in Chile that can give emotional and cognitive support to those caregivers of ASD people that need it. Thereby, they are not ignored, and it is not assumed that they are capable of carrying out labor that requires a total adjustment of their lifestyle.

## References

[1] World Health Organization (2023). Autism [Internet].

[2] Rojas V, Rivera A, Nilo N (2019). Actualización en diagnóstico e intervención temprana del Trastorno del Espectro Autista. Rev Chil Pediatr.

[3] Centers for Disease Control and Prevention Data and statistics on autism spectrum disorder [Internet].

[4] Ministerio de Salud (2011). Guía de práctica clínica: de detección y diagnóstico oportuno de los trastornos del espectro autista (TEA) [Internet].

[5] Abidin RR (2012). Parenting stress index – PSI-4.

[6] Fernández Suarez MP, Espinoza Soto AE (2019). Salud mental e intervenciones para padres de niños con trastorno del espectro autista: una revisión narrativa y la relevancia de esta temática en Chile. Revista de Psicología.

[7] Whitmore KE (2016). Respite care and stress among caregivers of children with autism spectrum disorder: an integrative review. J Pediatr Nurs.

[8] Segovia Brunschwig CI (2018). Estudio de la relación entre el nivel de integración de la estructura de personalidad, el estrés parenteral y la sintomatología depresiva en padres y madres de niños/as de 1 a 3 años [thesis].

[9] Lazarus RS, Folkman S (1986). Estrés y procesos cognitivos.

[10] Barquín-Cuervo R, Medina-Gómez MB, Albéniz-Garrote GP (2018). El uso de estrategias de afrontamiento del estrés en personas con discapacidad intelectual. Psychosocial Intervention.

[11] Barenghi FS (2020). Crianza parental y estrategias de afrontamiento frente al estrés en padres de niños con autismo [thesis].

[12] García R, Irarrázaval M, López I, Riesle S, Cabezas M, Moyano A (2021). Encuesta para cuidadores de personas del espectro autista en Chile: primeras preocupaciones, edad del diagnóstico y características clínicas. Andes Pediátrica.

[13] López-Chávez C, Larrea-Castelo ML, Breilh J, Tillería Y (2020). La determinación social del autismo en población infantil ecuatoriana. Rev Cienc Salud.

[14] Martínez Martín MA, Bilbao-León MC (2008). Acercamiento a la realidad de las familias de personas con autismo. Psychosocial Intervention.

[15] González MN, Depaula PD (2023). Parenting stress and coping strategies in mothers with children with Attention Deficit Hyperactivity Disorder. Revista Argentina de Ciencias del Comportamiento.

[16] Perez Padilla J, Álvarez-Dardet SM, Victoria Hidalgo M (2014). Estrés parental, estrategias de afrontamiento y evaluación del riesgo en madres de familias en riesgo usuarias de los Servicios Sociales. Psychosocial Intervention.

[17] Gadea AM, Miranda BR, Forner CB, Fortea IB, Casas AM (2020). Comparative study of stress, coping strategies and social support in mothers of children with autism spectrum disorder without intellectual disability and children with typical development. Rev Mex Psicol.

[18] Martínez-Montilla JM, Amador-Marín B, Guerra-Martín MD (2017). Estrategias de afrontamiento familiar y repercusiones en la salud familiar: una revisión de la literatura. Enferm Glob.

[19] Ávila-Beltrán F, Soliz H (2006). Impacto psicosocial del autismo en la familia. Gac Méd Boliv.

[20] Lee JZ, Iverson JM, Roemer EJ, Plate S, Schneider JL (2021). "I'm worried about my child": a longitudinal investigation of parental concerns and repeat screening in toddlers with familial risk of autism spectrum disorder. Pediatrics.

[21] Cala Hernández O, Licourt-Otero D, Cabrera-Rodríguez N (2015). Autismo: un acercamiento hacia el diagnóstico y la genética. Rev Ciencias Médicas.

[22] Lampert-Grassi MP Trastorno del espectro autista. Epidemiología, aspectos psicosociales y políticas de apoyo en Chile, España y Reino Unido.

[23] Gobierno de Chile (2017). Académicos liderarán estudio que contabilizará la población con autismo en el país [Internet].

[24] Tardón L (2011). El riesgo genético del autismo, más alto de lo que se pensaba [Internet].

[25] Bardají BM (2019). Comunicación y lenguaje en el TEA [Internet].

[26] Constantino JN, Kennon-McGill S, Weichselbaum C, Marrus N, Haider A, Glowinski AL (2017). Infant viewing of social scenes is under genetic control and is atypical in autism. Nature.

[27] Barrera Carmona N, Gutiérrez Moctezuma J (2004). Efecto de la risperidona en la modificación de la conducta y estereotipias en el paciente con trastorno autista. Rev Mex Neuroci.

[28] Paula-Pérez I, Artigas-Pallarés J (2016). Vulnerabilidad a la autolesión en el autismo. Rev Neurol.

[29] Damas A, Morillo A (2017). Sistema familiar y estrategias de afrontamiento en padres de niños con autismo. Psiquis UBA [Internet].

[30] Cid García T (2017). Rasgos depresivos y estrategias de afrontamiento en cuidadores de personas con autismo [thesis].

